# Spiritual Complexity in Palliative Home Care in Spain: A Multi-center Prospective Study

**DOI:** 10.1007/s10943-025-02300-y

**Published:** 2025-04-07

**Authors:** Xavier Busquet-Duran, Eduard Moreno-Gabriel, Maria Verdaguer, Eva Maria Jiménez-Zafra, Josep Maria Manresa-Domínguez, Pere Torán-Monserrat

**Affiliations:** 1 Home Care Programme Support Team (PADES) Granollers, Catalan Health Institute, Granollers, Spain; 2 Department of Social Psychology, Universitat Autònoma de Barcelona, Bellaterra (Cerdanyola del Vallès), Spain; 3 Research Support Unit North Metropolitan Area, Primary Care Research Institute Jordi Gol (IDIAPJGol), Mataró, Spain; 4 Multidisciplinary Research Group in Health and Society (GREMSAS) (2021-SGR-01484), Mataró, Spain; 5 Department of Medicine, Faculty of Medicine, Universitat de Girona, Girona, Spain

**Keywords:** Spiritual care, Existential suffering, Palliative care, Home-care, Complexity

## Abstract

This study examined spiritual complexity in end-of-life patients cared for by palliative care teams in Catalonia, Spain, using the HexCom model. Among 1818 patients (55.9% men, average age 75.7), spiritual complexity remained stable (37.5% initially, 35.5% final), while high complexity increased from 8.3 to 11.2%. Intrapersonal complexity was the most common (19.7%), followed by transpersonal (18.4%), and interpersonal (6.8%). Emotional complexity was strongly correlated with spiritual complexity. Key factors included cognitive impairment as a protector and how spiritual complexity sub-areas relate to desires to hasten death, family relationships, and end-of-life circumstances. The findings emphasize integrating spiritual care into routine interdisciplinary care.

## Introduction

Suffering is defined as the feeling of helplessness in the face of a threat (e.g., Chapman & Gavrin, 1999). This feeling is always modulated by one’s state of mind. It is also influenced by the perception of changes, both internal and external (Bayés et al., 1996). Palliative care aims to alleviate the suffering of people on the verge of death. In this situation, the psychological, social, and spiritual aspects of suffering must be considered as important as the biological ones (Sallnow et al., 2022). Often, however, they will present together and be inseparable, because it is not the bodies suffering, but the people (Cassell & Rich, 2010).

In the 1960s, palliative care pioneer Cicely Saunders began to promote the concept of “total pain” to articulate how end-of-life suffering is not only physical but also psychological, social and spiritual (Clark, 2016). Notwithstanding some unintended consequences of Saunders’ conceptualization, it timelessly highlighted the multifaceted character of the suffering specific to a life-limiting illness, including the spiritual dimension (Rattner, 2023).

According to Best et al. (2020), spirituality is a dynamic aspect of human life that relates to different aspects. First, it refers to the way people (individual and community) experience, express and/or seek meaning, purpose, and transcendence. Also, it relates to the way they connect with the moment, themselves, others and nature. Finally, it has to do with what is significant and/or sacred. In clinical practice, spirituality can emerge as a coping resource for situations that threatens the integrity of the self (Christie et al., 2023). In this regard, it can favor a person’s sense of control and it may have a positive effect on physical and emotional symptoms (Barreto et al., 2015; Delgado-Guay et al., 2016). Moreover, spirituality correlates statistically with resilience (Redondo Elvira et al., 2017).

In a study interviewing 57 patients with terminal cancer about their experiences and intensity of spiritual pain, 96% expressed having felt *spiritual pain* at some point in their lives and 61% at the time of the interview (Mako et al., 2006). At the beginning of the interviews, these authors described spiritual pain to the participants as “a deep pain in the soul that is not physical; a pain manifested as conflict” (p. 1108). Drawing on a content analysis of the interviews, the authors went on to classify patients’ descriptions of their spiritual pain following Pargament et al. (2006) three dimensions: intrapsychic, interpersonal and the divine. Likewise, we understand that human spirituality can be split into three dimensions, nonetheless we prefer the term “transpersonal” to refer to the need for control and security, and the need to trust and leave a legacy (Benito et al., 2014b). Besides, these dimensions contain a balanced part and an updated part, and these are oriented both to sustain the current situation and to face changes that will come (Benito et al., 2014a).

The care of spirituality in our techno-scientific context, where there is a clear weighting toward the physiological and somatic, is a challenge for health professionals. Although they perceive spirituality as a very important area of ​​need, they often feel incompetent when dealing with it (Dones Sánchez et al., 2016). Several factors contribute to this. These include lack of training and preparation (Bernard et al., 2017), the realization that some religious practices and elements can negatively influence the individual (Santos et al., 2022), and a secularist bias that complicates the ability to read religious and spiritual care needs (Dellenborg & Enstedt, 2023). This explains the lack of systematized protocols in clinical practice (Rudilla et al., 2018). Moreover, Europe’s secularized context, with high agnosticism and cultural and religious diversity, demands a broad view of spirituality, extending beyond religious aspects (Balboni et al., 2022).

In order to mitigate some of these limitations, most of palliative home care teams in Catalonia (northeast Spain) are multidisciplinary. These are made up mainly of medical, nursing, social work, and psychology professionals (Doblado et al., 2016). They are professionals specifically trained in palliative care, even though many of them feel their skills for providing spiritual care are scarce (Dones Sánchez et al., 2016). Besides, many of these teams are increasingly adopting holistic and integrative models such as the HexCom model (Busquet-Duran et al., 2020) to approach patients, including a systematic approach to spiritual complexity in palliative home care, considering the three dimensions defined above (intra-, inter-, and transpersonal). This model defines complexity as the difference between the needs of patients and the ability of health services to respond to them. Therefore, what is important for healthcare practice is not so much the symptom that the person suffers from or the suffering that the symptom generates, but the difficulty that professionals face in meeting and managing this need (Grembowski et al., 2014). In addition to the spiritual area, it includes the areas of clinical, psychological, socio-familial, and ethical needs, and those related to death. It also includes the strengths of the system that can sustain the situation (see Table 1 for descriptions of each area and strength).

**Table 1:** Definitions of the different sub-areas of needs and strengths of the HexCom model

Area	Sub-area	Definition
*Needs*		
Clinical	Physical	Physical discomfort due to symptoms (pain, dyspnea, etc.) and/or injuries (tumor ulcer, etc.)
	Therapeutic	Difficulty in adhering to prescriptions and/or accessing drugs/techniques
Psycho-emotional	Personality	Psychological vulnerability: rigid personality traits with difficulty adapting to changes (perfectionism, thoroughness, control, etc.), or psychopathology (alcoholism, drug addiction, psychiatric illness, dementia with behavioral changes, delirium, etc.)
	Emotional	Maladaptive emotional distress (intense, persistent, interfering with relationships and functioning)
Spiritual	Meaning	Deep distress with a feeling of being broken by the illness, with difficulty finding meaning in the situation, a feeling of inconsistency with the actions and decisions taken throughout life
	Connection	Deep distress with isolation and relational breakdown, feelings of guilt, not feeling at peace with others or not feeling part of them, difficulty forgiving, lack of insight
	Transcendence	Deep distress with difficulty facing everything that will happen, facing the unknown: panic about dying, disappearing, panic about the future of those left behind, difficulty seeing what will be left behind, feelings of injustice
Social and family	Relational	Relational distress in the family environment that makes patient care difficult
	Emotional	Maladaptive emotional distress of the carer/s (intense, persistent, that makes relationships and functioning difficult) and which makes it difficult to care for the patient
	Practice	Distress due to difficulties in handling the patient’s basic needs (hygiene, food, safety, etc.)
	External	Distress due to lack of effective external support in the living environment
	Economic	Financial distress and/or difficulty hiring external help and/or accessing resources
Ethics	Information	Distress due to difficulties in handling information about the diagnosis and/or prognosis
	Clinical decisions	Difficulties in making clinical decisions (adequacy of diagnostic and/or therapeutic effort)
	Desire to bring forward death	Desire to bring forward death to any degree: thought, intention, decision, plan and/or explicit request
Related directly with death/process of dying	Location	Difficulties in planning the place to die (no agreement between patient-caregiver) or request to change location
	Last days circumstances	Difficulties in managing the dying process (maladaptive denial of the situation, refractory symptoms, difficult sedation, etc.)
	Bereavement	Risk factors in bereavement
*Strengths*		
Micro-system	Individuals: the patient and their carers	The patient’s strength (values, mental capacity, resilience/adaptability, preferences, decisions, etc.)
		The strength of family caregivers (values, mental/physical capacity, resilience/adaptability, extensive network, etc.)
Meso-system	Interactions: patient interaction with the relational network and the professional network	The strength of family and/or community ties (tone of caring, ability to organize, etc.)
		The strength of the links with the referring professionals (doctor and/or head nurse, specialist, etc.)
		Economic strength
Chrono-system	Time: changes in time	Having in order pending issues that generate concern (inheritance, financial issues, inheritances, ties)
		Anticipating a progressive course for the disease, without sudden changes, and without changes in the caring environment
Exosystem	Team: the team's competence	The strength and competence of the team to care for the needs of the patient/family (interdisciplinary, training, communication, integration in the community, continuity and consensus on the therapeutic goal)
Macrosystem	Resources: availability of health and socio-health services/resources	Sufficient funding and accessibility to services and/or socio-sanitary resources to meet the patient’s needs (continuity of care, home help service, dependency law, residential places, hospital or socio-sanitary admission, radiotherapy units, pain clinic, day hospital, etc.)

We are not aware of any studies that specifically delve into spiritual complexity in palliative home care in European and Mediterranean contexts. Hence, we set out to study our hypothesis that healthcare professionals working in Catalan palliative home care perceive spiritual pain in at least one in three patients. Additionally, we expect that the three dimensions of spirituality will be characterized differently.

### Aims

(1) To investigate the prevalence of spiritual complexity at the time of starting care and up to death; (2) To understand the relationship between spiritual complexity and socio-demographic, health, and welfare variables and complexity variables; and (3) To investigate the differences between the three dimensions of spirituality.

## Material and Methods

### Design

Longitudinal observational study of a multicenter cohort.

### Scope

Specialized palliative home care in Catalonia (northeast Spain). The study included 76% of the 59 Home Care Support Teams (*PADES*) existing at that time (Busquet-Duran et al., 2023).

### Randomization

Consecutive recruitment was conducted during the study period (January 2016 to December 2020).

### Participants

The cohort included 1818 patients. The inclusion criteria were the presence of an advanced and/or end-of-life illness and being cared for by a *PADES* team. Patients who received care from nursing homes were excluded.

### Outcome Measures

The dependent variable was the level of overall spiritual complexity of each of the sub-areas (meaning, connection, and transcendence), assessed as high, moderate, low, or not assessable. The global grading was the highest in the sub-areas. The assessment was carried out by consensus among all the professionals in the team, and it was categorized according to the degree of difficulty it represented, after the first home assessment, and after the patient’s death.

The following were considered as independent variables: socio-demographic data (age, sex, relationship with the caregiver, support from family worker), pathologies (medical diagnosis (ICD-10), and groups of advanced diseases: cancer, advanced chronic organ failure, advanced neurological disease, dementia, or geriatric frailty/multimorbidity), the diagnosis that most contributes to the death process (by team consensus), mental status (Pfeiffer test), functional status (Barthel index), being bedridden: Palliative Performance Status PPS (Sancho Zamora et al., 2014), level of therapeutic intensity (Fontecha-Gómez et al., 2018), number of days of *PADES* care, whether they received psychosocial care, and place of death. The remaining 15 sub-areas of need and, from 2020, the 9 sub-areas of strength were included (Table 1). Both high and moderate complexity require the intervention of a specialized team; therefore the dichotomous variable “high/moderate complexity” versus “low or unrated complexity” was established for the statistical analysis.

### Data Collection

All variables were assessed using the HexCom instrument in an interdisciplinary manner after the first home visit, and after death.

Complexity is graded into three levels: high (a refractory situation, perceived as almost impossible to change), moderate (a difficult situation, which requires sharing the case), and low (an easy situation to manage). This model has been widely validated (Busquet-Duran et al., 2021a, 2021b, 2022; Esteban-Pérez et al., 2018) and is considered to offer the most extensive determinations of complexity (Grant et al., 2021). Spiritual complexity within HexCom is defined as deep distress with a sense of biographical rupture, lack of meaning (personal, vital, of suffering), loneliness (unwanted, isolation due to relational breakdown), feelings of guilt, impossibility of forgiveness (for oneself, for others), panic about the future (own or loved ones), and/or feelings of injustice.

To standardize data collection and compilation, participating teams received 10 h of face-to-face training, a user guide, and were provided with a phone number to call in case of questions during fieldwork.

### Statistical Analysis

Qualitative variables are summarized with their absolute and relative frequencies. Pearson’s Chi Square test and McNemar’s test were used in the case of analyzing paired data. Based on the cohort of included patients, the database was reviewed to purify it and analyze its quality, and a multivariate logistic regression analysis was performed to determine which factors were associated with spiritual complexity. For this reason, the outcome variable was divided into “high/medium spiritual complexity” and “low/not rated spiritual complexity”. The following were also dichotomized: age with a cutoff point of 70 years or more, functional status at a cutoff point of < 40 (total dependence, severe, according to the Barthel test), cognitive impairment at a cutoff point ≥ 3 (mild, moderate, severe impairment according to the Pfeiffer test), and care days at 7 days or more. In the initial assessment, the analysis was adjusted for age and sex, and in the final assessment, for age, sex, and care days. Initially, a saturated model was considered, with all variables showing a significant association in bivariate analysis. Next, those that were not significant were eliminated step-by-step (backward stepwise regression). The significance level was set at 5% for all contrasts.

### Ethical Considerations

The study complies with the principles of the Declaration of Helsinki and applicable regulatory requirements in ethical and data protection aspects. All participants read and signed an informed consent form. The study was approved by the Clinical Research Ethics Committee of the institution where researchers worked (P15/171) and by the clinical research ethics committees of all participating centers.

## Results

### Cohort

Overall, 1818 patients were recruited, with a predominance of men (55.9%) and an average age of 75.74 years (SD 13.17), very often with their partner as a caregiver (48.6%) (Table 2) Globally, they showed moderate dependence (Barthel Index of 58 average), 28.2% were bedridden at the first visit, 31.7% had cognitive impairment, 74.6% had an oncological pathology, and the level of therapeutic intensity was conservative in 76.7% of the cases. They were cared for for a median of 45 days, 32.3% underwent psychosocial assessment, and 51.4% died at home. The main sources of complexity in the initial assessment were socio-familial (53.1% due to lack of support from outside the family), clinical (39.4% due to difficulty in handling physical discomfort), and emotional (35.7% due to difficulty in handling the patient’s emotional discomfort). In terms of strengths, the main one was personal ties (80.79%).

**Table 2:** Participant Demographic Characteristics (*N* = 1818) and Grading of spiritual complexity (univariate analysis)

		Grading of spiritual complexity in the initial assessment				
Variables	*N* = 1818	Not assessable	Low	Moderate	High	*P*
*Socio-demographic data*						
Age (mean and SD)	75.74 (13.17)	80.37 (12.91)	78.02 (12.46)	71.81 (13.04)	70.32 (13.35)	< 0.001
Sex female	801 (44.1%)	74 (54.0%)	455 (45.5%)	205 (38.7%)	67 (44.1%)	0.008
Caregiver partner	883 (48.6%)	51 (37.2%)	443 (44.4%)	306 (57.8%)	83 (54.6%)	< 0.001
Family worker	489 (28.1%)	52 (38.8%)	283 (29.7%)	125 (24.3%)	29 (20.9%)	< 0.001
Functional status (Barthel average and SD)	58.04 (35.38)	21.50 (31.73)	56.13 (36.53)	69.25 (27.83)	64.44 (30.19)	< 0.001
Bedridden (PPS < = 40)	448 (28.2%)	84 (69.4%)	256 (30.0%)	77 (15.9%)	31 (24.2%)	< 0.001
Cognitive impairment (Pfeiffer > 2 errors)	501 (31.7%)	95 (77.9%)	315 (37.1%)	67 (13.9%)	24 (18.9%)	< 0.001
Disease type: cancer	1357 (74.6%)	53 (3.9%)	716 (52.8%)	458 (33.8%)	130 (9.6%)	< 0.001
Therapeutic intensity: conservative^a^	743 (76.7%)	43 (89.6%)	416 (78.3%)	238 (73.2%)	46 (70.8%)	0.06
Care days (median and quartiles)	45 (14–129)	16 (5–69)	46 (14–129)	54 (20–150)	40 (15–112)	0.020
Psychosocial assessment	587 (32.3%)	24 (17.5%)	268 (26.8%)	243 (45.8%)	52 (34.2%)	< 0.001
Death at home	831 (51.4%)	81 (65.3%)	473 (54.4%)	221 (46.0%)	56 (38.9%)	< 0.001
Clinical physical (difficulty in managing symptoms/injuries)^b^	716 (39.4%)	49 (35.8%)	302 (30.2%)	269 (50.8%)	96 (63.2%)	< 0.001
Clinical therapeutic (low commitment/difficulty accessing drugs/techniques)^b^	431 (23.7%)	35 (25.5%)	166 (16.6%)	170 (32.1%)	60 (39.5%)	< 0.001
Psychological personality (psychological vulnerability)^b^	553 (30.4%)	62 (45.3%)	274 (27.5%)	147 (27.7%)	70 (46.1%)	< 0.001
Psychological emotional (maladaptive emotional distress)^b^	649 (35.7%)	42 (30.7%)	129 (12.9%)	342 (64.5%)	136 (89.5%)	< 0.001
Socio-familial relationships (family relational distress)^b^	195 (10.7%)	10 (7.3%)	60 (6.0%)	74 (14.0%)	51 (33.6%)	< 0.001
Socio-familial emotional (Caregiver maladaptive emotional distress)^b^	716 (39.4%)	54 (39.4%)	307 (30.7%)	262 (49.4%)	93 (61.2%)	< 0.001
Socio-familial practice (difficulty handling basic patient needs)^b^	483 (26.6%)	41 (29.9%)	190 (19.0%)	183 (34.5%)	69 (45.4%)	< 0.001
Socio-familial external (lack of effective external support for core coexistence)^b^	965 (53.1%)	74 (54.0%)	468 (46.8%)	317 (59.8%)	106 (69.7%)	< 0.001
Socio-familial economic (financial discomfort/difficulty hiring help)^b^	274 (15.1%)	20 (14.6%)	118 (11.8%)	97 (18.3%)	39 (25.7%)	< 0.001
Ethics information (difficulty in handling diagnostic/prognostic information)^b^	174 (9.6%)	6 (4.4%)	72 (7.2%)	70 (13.2%)	26 (17.1%)	< 0.001
Ethics (therapeutic adequacy (difficulty making clinical decisions)^b^	289 (15.9%)	29 (21.2%)	129 (12.9%)	91 (17.2%)	40 (26.3%)	< 0.001
Ethics desire to bring forward death (desire to bring forward death to any degree)^b^	79 (4.3%)	0 (0.0%)	19 (1.9%)	29 (5.5%)	31 (20.4%)	< 0.001
Death location (difficulty in planning the place to die)^b^	1116 (61.4%)	68 (49.6%)	534 (53.5%)	396 (74.7%)	118 (77.6%)	< 0.001
Death situation of last days (difficulty handling situation of last days)^b^	38 (2.1%)	5 (3.6%)	18 (1.8%)	11 (2.1%)	4 (2.6%)	0.52
Death bereavement (presence of bereavement risk factors)^b^	632 (34.8%)	39 (28.5%)	273 (27.3%)	246 (46.5%)	74 (48.7%)	< 0.001
Patient (the strength of the patient)^c^	414 (44.3%)	1 (2.2%)	233 (45.8%)	160 (50.5%)	20 (31.7%)	< 0.001
Caregiver (the strength of family caregivers)^c^	632 (67.7%)	23 (51.1%)	375 (73.7%)	206 (65.0%)	28 (44.4%)	< 0.001
Ties family (the strength of family and/or community ties)^c^	756 (80.9%)	32 (71.1%)	439 (86.2%)	245 (77.3%)	40 (63.5%)	< 0.001
Ties professional (the strength of ties with relevant professionals)^c^	704 (75.4%)	24 (53.3%)	416 (81.7%)	234 (73.8%)	30 (47.6%)	< 0.001
Economic (economic strength)^c^	643 (68.8%)	31 (68.9%)	365 (71.7%)	211 (66.6%)	36 (57.1%)	0.081
Pending issues (having sorted pending issues that were worrying)^c^	580 (62.1%)	23 (51.1%)	372 (72.1%)	162 (51.1%)	23 (36.5%)	< 0.001
Progressive course (predicting a progressive course of the disease, without sudden changes, and without changes in the caring environment)^c^	298 (31.9%)	18 (40.0%)	206 (40.5%)	59 (18.6%)	15 (23.8%)	< 0.001
Exosystem (the strength/competence of the team to care for the needs of the patient/family)^c^	838 (89.7%)	32 (71.1%)	468 (91.9%)	289 (91.2%)	49 (77.8%)	< 0.001
Macrosystem (sufficient funding and accessibility to services and/or socio-health resources to meet the patient’s needs)^c^	800 (85.7%)	29 (64.4%)	440 (86.4%)	279 (88.0%)	52 (82.5%)	< 0.001

^a^Assessment introduced from 2020. ^b^Complexity M + H: Number of patients with medium or high complexity of a certain need. ^c^ Strength A: Number of patients with high strength in a given subsystem

### Global Spiritual Complexity

The prevalence of global spiritual complexity, namely without distinguishing whether it was intrapersonal, interpersonal or transcendental, was 37.5% at the initial assessment and 35.5% at the final assessment (Table 3). The prevalence of high complexity, assessed as potentially refractory, increased from 8.3 to 11.2% (*p* = 0.001).

**Table 3:** Evolution of the global spiritual complexity, at the beginning of care and after death

Degree of global spiritual complexity (*N* = 1818)				
Assessment	Not assessable	Low	Medium	High
Initial	137 (7.53%)	999 (54.95%)	530 (29.15%)	152 (8.36%)
Final	134 (7.3%)	1039 (57.2%)	441 (24.3%)	204 (11.2%)

Multivariate logistic regression analysis (Table 4) showed a strong relationship between initial global spiritual complexity and difficulty in handling patients’ emotional distress (OR 15.62), desire to hasten death (OR 4.85), and physical discomfort (OR 1.74). It showed an inverse relationship with cognitive impairment (OR 0.30), having a perspective of the progressive course of the disease (OR 0.52), being hospitalized at the first visit (OR 0.53), and being over 70 years old (OR 0.61). In the final assessment, the correlations between difficulties in handling information (OR 3.86) and difficulties in handling family relationships (OR 2.47) were strong, and only cognitive impairment showed an inverse relationship (OR 0.29). Finally, we analyzed the differences within those who presented spiritual complexity but not emotional complexity (*N* = 204). Interestingly, the only difference that was found was that, out of these patients, 43.4% received psychosocial support, while among those who only presented emotional complexity, this support was given in 27.2% of cases (*p* = 0.003).

**Table 4:** Global spiritual complexity. Results of the multivariate analysis of the determining factors of complexity in the initial assessment (adjusted for age and sex) and final assessment (adjusted for age, sex and care days)

Initial GLOBAL spiritual complexity				Final GLOBAL spiritual complexity			
Variables	Beta	OR (CI95%)	*P*	Variables	Beta	OR (CI95%)	*P*
Age = / > 70	− 0.501	0.61 (0.41; 0.90)	0.014	Age = / > 70	− 0.244	0.78 (0.58; 1.07)	0.122
Sex: female	− 0.313	0.73 (0.51; 1.06)	0.096	Sex: female	− 0.128	0.88 (0.65; 1.20)	0.415
				Care days > 7	0.519	1.68 (1.07; 2.64)	0.024
				Caregiver partner	0.279	1.32 (0.97; 1.80)	0.076
Bedridden^a^	− 0.632	0.53 (0.34; 0.84)	0.007				
Psychosocial assessment	0.605	1.83 (1.27; 2.64)	0.001				
Cognitive impairment^b^	− 1.219	0.30 (0.19; 0.47)	< 0.001	Cognitive impairment^b^	− 1.226	0.29 (0.20; 0.43)	< 0.001
Physical distress^c^	0.557	1.74 (1.20; 2.53)	0.003	Physical distress^c^	0.602	1.82 (1.37; 2.44)	< 0.001
Emotional distress^c^	2.749	15.62 (10.44; 23.37)	< 0.001	Emotional distress^c^	2.696	14.8 (10.9; 20.2)	< 0.001
				Psychological vulnerability^c^	− 0.275	0.76 (0.54; 1.07)	0.111
Basic needs^c^	0.550	1.73 (1.12; 2.68)	0.014				
				Relational distress^c^	0.903	2.47 (1.59; 3.82)	< 0.001
				Lack of external network^c^	0.361	1.43 (1.07; 1.92)	0.016
Desire to bring forward death^c^	1.580	4.85 (1.82; 12.94)	0.002	Desire to bring forward death^c^	1.408	4.09 (1.87; 8.93)	< 0.001
				Handling information^c^	1.351	3.86 (2.06; 7.23)	< 0.001
Progressive course forecast^d^	− 0.653	0.52 (0.34; 0.79)	0.002				
Constant	− 0.827			Constant	− 2.284		

^a^PPS Palliative Performance Status < = 40, ^b^Pfeiffer test > 2 errors. ^c^(M + H): Moderate or high complexity of the need. ^d^(H): High strength

### Intrapersonal Spiritual Complexity (Meaning)

In the initial assessment, complexity was detected in the intrapersonal dimension in 541 participants (19.7%). The multivariate logistic regression analysis (Table 5) showed a strong relationship with difficulties in handling emotional distress (OR 12.58) and with the desire to bring forward death (OR 4.22), and an inverse relationship with cognitive impairment (0.34), being already hospitalized at the first visit (0.50), or having a perspective on the progressive course of the disease (0.59). In the final assessment, difficulty in handling emotional distress had a lower weight (OR 7.9), and only difficulty in handling external family support showed an inverse relationship (OR 0.17).

**Table 5:** Intrapersonal spiritual complexity

Intrapersonal spiritual complexity initial meaning				Intrapersonal spiritual complexity final meaning			
Variables	Beta	OR (CI95%)	*P*	Variables	Beta	OR (CI95%)	*P*
Age = / > 70	− 0.204	0.82 (0.55; 1.21)	0.306	Age = / > 70	0.232	1.26 (0.86; 1.86)	0.238
Sex: female	− 0.197	0.82 (0.57; 1.18)	0.283	Sex: female	0.174	1.19 (0.82; 1.72)	0.356
				Care days > 7	1.034	2.81 (1.20; 6.62)	0.018
Bedridden^a^	− 0.702	0.50 (0.31; 0.78)	0.003				
Cognitive impairment^b^	− 1.086	0.34 (0.22; 0.53)	< 0.001				
Physical distress^c^	0.639	1.89 (1.32; 2.72)	0.001				
Emotional distress^c^	2.532	12.58 (8.69; 18.23)	< 0.001	Emotional distress^c^	2.062	7.9 (5.1; 12.0)	< 0.001
Basic needs^c^	0.783	2.19 (1.43; 3.35)	< 0.001				
				Lack of external network^c^	− 1.768	0.17 (0.10; 0.29)	< 0.001
Desire to bring forward death^c^	1.439	4.22 (1.73; 10.31)	0.002				
Progressive course forecast^d^	− 0.532	0.59 (0.39; 0.89)	0.013				
Team strength^d^	0.809	2.25 (1.24; 4.06)	0.007				
Constant	− 2.023			Constant	− 4.220		

Results of the multivariate analysis of the determining factors of complexity in the initial assessment (adjusted for age and sex) and final assessment (adjusted for age, sex and care days)

^a^PPS Palliative performance status < = 40, ^b^Pfeiffer test > 2 errors. ^c^(M + H): Moderate or high complexity of the need. ^d^(H): High strength

### Interpersonal Spiritual Complexity (Connection)

In the initial assessment, complexity was detected in the interpersonal dimension in 124 (6.8%) patients. Logistic regression analysis (Table 6) showed a strong relationship, in the initial assessment, with difficulties in managing family relationships (OR 20.4), emotional distress management (OR 5.10) and external support network management (OR 3.93). Conversely, it only showed an inverse relationship with information handling (OR 0.24). The final assessment interpersonal spiritual complexity was mainly related to family relational distress (OR 16.7), the strength of the patient (2.16) and the complexity due to emotional distress, which increased considerably (OR 13.4), while the strength of family ties appeared as an inverse relationship (OR 0.29).

**Table 6:** Interpersonal spiritual complexity

Interpersonal spiritual complexity initial connection				Interpersonal spiritual complexity final connection			
Variables	Beta	OR (CI95%)	*P*	Variables	Beta	OR (CI95%)	*P*
Age = / > 70	− 0.117	0.89 (0.56; 1.42)	0.626	Age = / > 70	0.362	1.44 (0.68; 3.05)	0.345
Sex: female	− 0.171	0.84 (0.53; 1.33)	0.464	Sex: female	− 0.161	0.85 (0.41; 1.75)	0.662
				Care days > 7	0.120	1.13 (0.24; 5.23)	0.878
Death at home	0.002	1.00 (0.70; 1.44)	0.993	Death at home	− 1.026	0.36 (0.17; 0.78)	0.009
Emotional distress^c^	1.629	5.10 (3.14; 8.28)	< 0.001	Emotional distress^c^	2.593	13.4 (5.53; 32.3)	< 0.001
Relational distress^c^	3.017	20.4 (12.9; 32.2)	< 0.001	Relational distress^c^	2.817	16.7 (7.4; 37.8)	< 0.001
Lack of external network^c^	1.368	3.93 (2.11; 7.32)	< 0.001				
Handling information^c^	− 1.444	0.24 (0.10; 0.57)	0.001	Handling information^c^	− 1.523	0.22 (0.06; 0.81)	0.024
				Patient strength^d^	0.771	2.16 (0.99; 4.71)	0.053
				Strength of family ties^d^	− 1.252	0.29 (0.13; 0.65)	0.003
Constant	− 5.165			Constant	− 4.315		

Results of the multivariate analysis of the determining factors of complexity in the initial assessment (adjusted for age and sex) and final assessment (adjusted for age, sex and care days)

^a^PPS Palliative performance status < = 40, ^b^Pfeiffer test > 2 errors. ^c^(M + H): Moderate or high complexity of the need. ^d^(H): High strength

### Transpersonal Spiritual Complexity (Transcendence)

In the initial assessment, complexity was detected in the transpersonal dimension in 366 (18.4%) participants. Logistic regression analysis (Table 7) showed a strong relationship with difficulties in handling emotional distress (OR 14.47) and difficulties in handling basic needs (1.81). In contrast, many variables showed an inverse relationship: being already hospitalized at the first visit (OR 0.37), a level of conservative therapeutic intensity (OR 0.43), being over 70 years old (OR 0.45), difficulties derived from psycho-emotional vulnerability (OR 0.51), and lack of pending issues (OR 0.55). The final assessment, transpersonal spiritual complexity was more strongly related to emotional distress (OR 22.4), handling difficulties arising from the situation in the last few days (OR 3.08), to handling clinical decisions (OR 2.41), with the fact that the caregiver was the partner (OR 1.98), and with a good functional status (OR 1.88). Anticipating a progressive course (OR 0.39), difficulty in managing aspects related to personality (OR 0.54) and difficulties in managing external support were added as factors with an inverse relationship in the family nucleus (OR 0.59).

**Table 7:** Transpersonal spiritual complexity

Transpersonal spiritual complexity initial transcendence				Transpersonal spiritual complexity final transcendence			
Variables	Beta	OR (CI95%)	*P*	Variables	Beta	OR (CI95%)	*P*
Age = / > 70	− 0.797	0.45 (0.29; 0.69)	< 0.001	Age = / > 70	− 0.463	0.63 (0.39; 1.01)	0.055
Sex: female	− 0.033	0.97 (0.64; 1.47)	0.876	Sex: Female	0.190	1.21 (0.75; 1.96)	0.438
				*PADES* care days > 7	0.062	1.06 (0.48; 2.37)	0.880
Bedridden^a^	− 1.007	0.37 (0.21; 0.64)	< 0.001				
				Caregiver: partner	0.681	1.98 (1.22; 3.20)	0.005
				Total/severe dependence	0.633	1.88 (1.04; 3.40)	0.036
Therapeutic intensity conservative	− 0.856	0.43 (0.25; 0.71)	0.001	Therapeutic intensity conservative	− 1.312	0.27 (0.15; 0.49)	< 0.001
Emotional distress^c^	2.672	14.47 (9.32; 22.46)	< 0.001	Emotional distress^c^	3.110	22.4 (13.7; 36.8)	< 0.001
Psychological vulnerability^c^	− 0.669	0.51 (0.33; 0.80)	0.003	Psychological vulnerability^c^	− 0.614	0.54 (0.33; 0.88)	0.014
Basic needs^c^	0.595	1.81 (1.13; 2.92)	0.014				
				Lack of external network^c^	− 0.522	0.59 (0.38; 0.93)	0.024
				Clinical decision making^c^	0.878	2.41 (1.33; 4.36)	0.004
				Handling the last few days^c^	1.123	3.08 (1.69; 5.58)	<0.001
Having no pending issues^d^	− 0.598	0.55 (0.36; 0.84)	0.005				
				Progressive course forecast^d^	− 0.935	0.39 (0.22; 0.70)	0.002
Constant	− 0.750			Constant	− 1.981		

Results of the multivariate analysis of the determining factors of complexity in the initial assessment (adjusted for age and sex) and final assessment (adjusted for age, sex and care days)

^a^PPS Palliative performance status < = 40, ^b^Pfeiffer test > 2 errors. ^c^(M + H): Moderate or high complexity of the need. ^d^(H): High strength. ^e^Barthel < 40

## Discussion

This study explored spiritual complexity in a large cohort of end-of-life patients cared for at home by palliative care expert teams in Catalonia (Spain). The prevalence of global spiritual complexity was stable throughout the care period whereas. High complexity increased from 8.3 to 11.2% (*p* = 0.001). Intrapersonal complexity was the most prevalent (19.7%), followed by transpersonal complexity (18.4%) and interpersonal complexity (6.8%). Spiritual complexity was strongly related to emotional complexity. Intrapersonal complexity was mainly related to the desire to bring forward death (OR 4.22), interpersonal complexity to relational difficulties with the family (OR 16.7), and transpersonal complexity to the management of the circumstances of the last days (OR 3.08). The factors that influence spiritual complexity are manifold and pivot, to a greater or lesser extent, on all the areas included in the HexCom model.

### Prevalence of Spiritual Complexity

The prevalence of global spiritual complexity is lower than expected based on several studies, which show that *spiritual pain* is reported to be 44–52% in terminal cancer patients (Delgado-Guay et al., 2016, 2021), and up to 70% in onco-hematological patients (Maldonado et al., 2024). Usually this *pain* is classified as mild by the patients themselves (Delgado-Guay et al., 2011) and this may cause the professional to not detect it as complex to manage. In any case, we think that the detected spiritual distress is probably less than the real distress, due in part to the lack of specific training of professionals, to hidden complexity (Pask et al., 2018) and also due to the fact that we live in a cultural context where both death and spirituality are, in a way, taboo (Hvidt et al., 2019).

The prevalence of spiritual complexity considered refractory by professionals is very similar to the 10.5% found in the 4840 Canadian patients with terminal illness cared for at home, who claimed to be fighting with the meaning of life, according to Freeman et al. (2016). The similarity is remarkable, and even more so if we consider that in this study, the source of information is the patients themselves. These results can also be related to the prevalence of demoralization syndrome, which ranges from 13 to 18% (Robinson et al., 2015), and to that of the desire to bring forward death, which ranges from 6 to 10% (Busquet-Duran et al., 2021a, 2021b). Demoralization is inversely related to spirituality (Ghiggia et al., 2021), and the desire to bring forward death is related to the three dimensions of spirituality, in an activating way regarding lack of meaning (OR 3.25) or connection (OR 3.81) and in a protective way regarding transcendence (OR 0.50) (Busquet-Duran et al., 2021a, 2021b).

### Emotional Distress

Our results show that there are clear lines of communication between spirituality and the emotional sphere. This is because suffering is always modulated by mood (Maté et al., 2009) and simultaneously influenced by the perception of change. The patients included in the cohort, the majority of whom had cancer, were in a situation of rapid functional decline, passing a few weeks or days from leading a practically normal life to being bedridden, with high functional dependence and close to death, a closeness that until then, has often been denied. This overlap between the emotional and spiritual justifies patients with strong spiritual distress being evaluated, at least in the first instance, by psychology professionals, as shown by the fact that of those who presented spiritual but not emotional complexity (*N* = 204), 43.4% received psychosocial support (*p* = 0.003).

### Interrelated Complexity Variables

Our results show that there is a nonlinear and dynamic interaction between most of the dimensions of needs explored in the HexCom model. In clinical practice, this means that, although we often face distress that cannot be modified directly, it can be mitigated by activating the protective factors that are related to it, especially in the transpersonal dimension, with multiple protective factors, especially in the socio-familial and ethical spheres. We must agree with Lormans (2021) when they state that social and spiritual needs show great similarities and overlap, and that without an in-depth exploration of the expression of these needs, it is difficult to differentiate them and assign appropriate health interventions. Likewise, the relationship between spiritual complexity and difficulties in communicating diagnosis and prognosis, suggests that we are in an environment where a conspiracy of silence is common, a phenomenon that greatly deteriorates relationships, obstructs the development of the spiritual dimension, and generates suffering in all actors involved, including professionals (García-Navarro et al., 2023). We think that a psycho-socio-spiritual and ethical approach is needed that honors reality, preserves the multidimensionality of patients’ needs, and allows for interdisciplinary teamwork to assign personalized and comprehensive care.

### Socio-Demographic Variables

The bond with the family caregiver and age are the only variables that seem to influence spiritual complexity, but only in the transpersonal dimension. The fact that the main caregiver is the partner can mean that the patient lives with concern for the future of those who will survive them. On the other hand, older people can take more responsibility for their own death. This result agrees with what was found in a sample of 1662 cancer patients, where it was seen how older people showed less *spiritual pain* (Christie et al., 2023). The inverted association between age and complexity, also observed in other studies (Carrasco-Zafra et al., 2020), may be due to the greater acceptance of death by the elderly and their families (Hodiamont et al., 2019). It has been seen that older people with cancer tend to report less negative psychosocial impact (Nipp et al., 2016) and less psychological distress (Acquati & Kayser, 2019).

### Limitations

These results can be extrapolated to the home care of people with terminal illnesses, predominantly cancers. This leads, in itself, to a selection bias, since people with relationship difficulties or serious coexistence problems, in general, are not cared for by these types of services but by admission units. This partly explains why interpersonal spiritual complexity is the least prevalent. The main limitation of this study is that the spiritual distress of the people cared for was not specifically contrasted through validated scales; therefore, it is not possible to determine the compatibility between this distress and the team’s difficulty in handling it. Additionally, assessing simultaneously spirituality and mental health can lead to so-called tautological association (Koenig & Carey, 2024), which could potentially explain the strong association between perceived spiritual and emotional complexities. Indeed, the distinction between spiritual and emotional discomfort in clinical practice is often difficult, making psychology professionals indispensable in palliative care teams. However, it must be stressed that HexCom is not a scale of assessment of the patient’s discomfort. More precisely, it is a measure of the difficulties professionals perceive when taking care of these dimensions.

As a strength, we have to consider the size of the sample, the fact that it is a multicenter prospective study with longitudinal data, and that it encompasses several rural and urban socio-demographic environments. It should also be noted that the assessment of complexity is carried out by the consensus of an interdisciplinary team, which always includes professionals from medicine, nursing, psychology, and social work. The intersubjectivity of the assessment can, in a certain way, objectify a phenomenon as subjective as existential suffering (Rodrigues et al., 2018). Likewise, it must be said that both the study and assessment of professionals are based on a clear and defined theoretical framework (the HexCom model) of a concept such as spirituality, which is often considered polysemic and controversial.

### Applicability

These results highlight the importance of spiritual care at the end of life. This is relevant because, in our strongly secularized cultural context, spiritual/religious and existential concerns are often taboo, and because health professionals struggle with understanding what is meant by spirituality or spiritual care; therefore, they often do not meet the spiritual needs of patients (Laranjeira et al., 2023).

Certainly, spiritual support is not a systematized practice in palliative care teams in the Spain. There are few teams that have a specific figure for this task and even fewer teams who have a common language and a system for the evaluation and care of spiritual needs. Moreover, there seems to be a tendency to equate spirituality and religion (Donés Sánchez et al., 2024). However, our results can raise awareness of these limitations and facilitate self-reflection among palliative care teams, considered a basic element of spiritual support (Benito Oliver & Rivera-Rivera, 2019). Finally, the results highlight the need for the integrated presence of people who are experts in spiritual support in teams, who are necessarily open lay people and knowledgeable about the practices surrounding death in different cultural and religious traditions (Wierstra et al., 2023). All of this is even more relevant at the current moment of deployment of the Euthanasia Law in our country if you consider that existential suffering may be the cause of 25% of continuous palliative sedation (Morita, 2004) and may be the origin of many requests for euthanasia or medically assisted suicide (Rodrigues et al., 2018).

### Lines of Future Research

In future studies, it would be necessary to incorporate specific scales that are answered by patients and include religious aspects and those on philosophy of life, as is currently being done in the pediatric field (Miquel et al., 2024). The integrative construction of religiosity, spirituality, and philosophy of life proposed by Hexem (2011) can provide a better conceptual framework to examine a context as secularized and as diverse as ours. In any case, this study wishes to contribute to research on community palliative care in general, a need that calls for recent systematic reviews (Vernon et al., 2022), and also wishes to contribute to the study of applicability of the complexity paradigm at the end of life (Hodiamont et al., 2019).

## Conclusions

In conclusion, the large number of variables related to spiritual complexity and its different roles, favoring or protective, require a holistic vision of care, far from standardizing protocols. Spiritual care should not be seen as separate from health care but must be integrated into the daily work of interdisciplinary teams. These teams must be trained in basic spiritual care and have professionals who are experts in spiritual care taking part in the dynamics of daily work.
